# Designing intelligent anesthesia for a changing patient demographic: a consensus statement to provide guidance for specialist and non-specialist anesthetists written by members of and endorsed by the Society for Obesity and Bariatric Anaesthesia (SOBA)

**DOI:** 10.1186/2047-0525-2-12

**Published:** 2013-06-06

**Authors:** Mark C Bellamy, Michael P Margarson

**Affiliations:** 1 St James’s University Hospital, Leeds, UK; 2 Department of Anaesthesia and Critical Care, St Richard’s Hospital, Chichester, West Sussex PO19 6SE, UK

## Abstract

**Background:**

As a result of the increasing prevalence of obesity in the UK, anesthetists are increasingly encountering overweight and obese patients in routine practice. There is currently a paucity of evidence to guide best clinical practice for anesthetists managing overweight and obese patients. The current guidelines from the Association of Anaesthetists of Great Britain and Ireland (AAGBI), entitled *Peri-Operative Management of the Morbidly Obese Patient*, give an excellent overview of organizational issues, but leave much clinical detail to the discretion of the individual clinician.

**Methods:**

In May 2010, a panel of experts convened to develop consensus on anesthesia of overweight, obese and morbidly obese patients, in consultation with the Society for Obesity and Bariatric Anaesthesia (SOBA). All Panel members are practicing clinicians from recognized bariatric surgical training centers and have extensive experience of anesthesia for obese patients. This statement aims to provide guiding principles on best practice for this challenging patient demographic, and to increase awareness of current issues so that these can be addressed more appropriately.

**Results:**

In this document, we emphasize key principles for best practice, rather than giving prescriptive guidance and specific regimens for all clinical eventualities. We provide evidence-based justification for best-practice techniques, where this exists. In areas for which there is no evidence, but there is clear consensus, we offer this as guidance. We also aim to dispel misconceptions that have arisen in the anesthetic practice of overweight, obese, and morbidly obese patients.

**Conclusion:**

Ultimately, the choice of specific technique depends on clinician experience, patient characteristics, and center facilities. As well as providing guiding principles for anesthesia, this consensus statement also highlights other areas where anesthetists can contribute towards the enhanced recovery and overall quality of patient care.

## Background

Owing to the increasing prevalence of obesity in the UK, anesthetists are increasingly encountering overweight and obese patients in routine practice. However, there is currently little clinical guidance for anesthetists in the management of this patient group. Therefore, in May 2010, a panel of experts convened to develop consensus on anesthesia of overweight, obese, and morbidly obese patients, in consultation with the Society for Obesity and Bariatric Anaesthesia (SOBA). All panel members are practicing clinicians from recognized bariatric surgical training centers, and have significant experience in anesthesia of overweight and obese patients.

### Changing patient demographic

**Figure 1:**
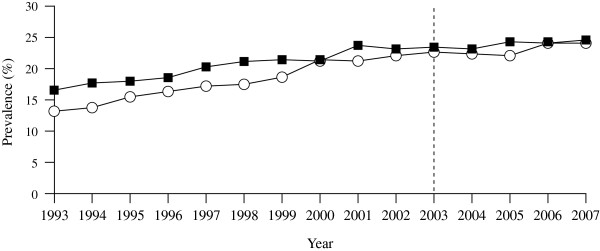
**The prevalence of adult obesity in England between 1993 and 2007.** Graphs show prevalence for women (■) and men (○). LEGEND: Data up to 2002 are unweighted; from 2003 onwards (dotted line shows cut-off point), data have been weighted for non-response. Adapted from the *Health Survey for England 2007 - Latest Trends* from The National Health Service Information Centre [1]. Copyright ©2012, republished with permission of The Health and Social Care Information Centre. All rights reserved.

Figure 1 shows the prevalence of obesity among adults in England between 1993 and 2007 [1]. The Foresight report, *Tackling Obesities: Future Choices*, estimated that 36% of men and 28% of women would be obese by 2015, and these figures would rise to 47% and 36%, respectively, by 2025 [2]. The Foresight report also estimated that the annual direct and indirect costs of obesity could reach £37 billion by 2025, consuming almost 12% of the total National Health Service (NHS) budget [2]. Recent US research shows that almost 17% of medical expenditure in the US goes towards the treatment of illnesses caused by obesity, almost double previous estimates [3]. Therefore, it is imperative that anesthetists become familiar with managing overweight and obese patients and understand the wider implications for the NHS.

### Lack of guidance in the field

There is currently a paucity of evidence regarding best clinical practice for anesthetists managing overweight and obese patients. The current guidelines from the Association of Anesthetists of Great Britain and Ireland (AAGBI), entitled *Peri-Operative Management of the Morbidly Obese Patient*, give an excellent overview of organizational issues, but leave much clinical detail to the discretion of the individual clinician [4].

### Aim and scope of this statement

This statement aims to provide guiding principles on optimal care for this changing patient demographic, and to increase awareness of current issues so that clinical challenges can be addressed more appropriately. In this document, we aim to emphasize key principles for best practice, rather than giving prescriptive guidance and specific regimens for all clinical eventualities. We provide evidence-based justification for best-practice techniques, where this exists. In areas for which there is no evidence, but there is clear consensus, we offer this as guidance. We also aim to dispel misconceptions that have arisen in anesthetic practice of overweight, obese, and morbidly obese patients. Ultimately, choice of the specific technique depends on clinician experience, patient characteristics, and center facilities.

### The enhanced recovery programme

As well as providing guiding principles for anesthesia, we hope that this consensus statement will highlight other areas in which anesthetists can contribute towards enhanced recovery and the overall quality of patient care.

The fundamental principles of best practice in anesthesia for overweight and obese patients are at the very heart of the Enhanced Recovery Programme:

•Better outcome and shortened length of stay for the patient, including early mobilization

•Structured approach for optimal pre-operative, peri-operative and post-operative care

•Reduction in the physiological stress of surgery.

Putting in place practices that are in alignment with these principles will deliver benefit both to individual patients and to the NHS as a whole. Because the healthcare needs of overweight and obese patients place a growing burden on the NHS, there is a clear need to bring clinical practice into alignment with the Enhanced Recovery Programme to focus on quality, improve productivity, eliminate waste, and curtail spiraling costs.

### Definition of obesity

The principles set out in this consensus statement apply according to: 1) the severity of obesity and 2) the physiological effects in terms of comorbidities. We will not address specific categories of obesity. However, it is useful to define classifications of overweight and obesity.

Body mass index (BMI) is the most common method of classifying adult weight. It is defined as weight in kilograms divided by the height in meters squared (kg/m^2^). Table 1 shows BMI ranges as defined by the WHO [5]. The medical literature gives further categories, including superobese (50 to 59.9 kg/m^2^), super-superobese (60 to 69.9 kg/m^2^) and hyperobese (>70 kg/m^2^) [6].

**Table 1:** **WHO international classification of adult overweight and obesity according to body mass index (BMI)**[5]

**Classification**	**BMI (kg/m**^**2**^**)**
Normal range	18.5–25
Overweight	≥25
Pre-obese	25–30
Obese	≥30
Obese class I	30–35
Obese class II	35–40
Obese class III (morbidly obese)	≥40

BMI is not an ideal measurement of obesity. It fails to take into account variations in body proportions in different populations. The WHO has investigated the need for developing different BMI cut-off points for definitions of obesity in different ethnic groups, including Asian and Pacific populations. A WHO Expert Consultation recommended additional cut-off points, which should be used in conjunction with the principal cut-off points in some populations [7].

Simple linear measurements, such as girth or neck circumference, are often more clinically relevant than BMI in measurement of obesity levels, because they may give a better idea of fat distribution. Consideration of fat distribution is very important, and although there is a whole spectrum of types of distribution, two major types are used for classification: android and gynecoid fat distribution, also knows as ‘apples’ and ‘pears’. Although the terms ‘android’ and ‘gynecoid’ refer to the typical male (centripetal) and female (peripheral) fat distributions, both distributions are seen in both genders.

The android type is of greater pathophysiological significance. It describes a physique in which the weight is carried on the trunk and there is a high intraperitoneal fat content. The patient is likely to have an increased neck circumference, but with little fat distributed on the arms and legs. A patient with an android fat distribution may have a modest BMI, of around 50 kg/m^2^, yet may have minimal physiological reserve and be at extreme risk of peri-operative death.

In a person with gynecoid fat distribution, more weight is carried on the arms, legs, and buttocks, and abdominal fat is predominantly extraperitoneal. Patients with very high BMIs will usually have this type of fat distribution and, paradoxically, may be at lower risk of mortality than those with an android fat distribution.

## Results and discussion

### Introduction to best-practice anesthetic techniques

A key principle for anesthesia is the employment of techniques that are familiar, reproducible, and safe. The anesthetist should not necessarily have to change practice just because the patient is larger than the average they encounter. Rather, a better outcome may be achieved by awareness of the issues that may arise in this population. The risk should not be exacerbated by incorporating new or unfamiliar techniques.

A second key principle is maintaining control and minimizing periods of potential risk or instability. In particular, care should be taken during the transition from spontaneous breathing to controlled ventilation during induction, and during the periods of emergence and extubation.

Techniques should incorporate Enhanced Recovery principles to provide optimal care.

### Premedication and preparation techniques

Antacid prophylaxis with histamine 2 antagonists or proton-pump inhibitors may be used as indicated, for example in patients with pre-existing, untreated reflux. This is often continued into the post-operative period. Pre-emptive analgesia with paracetamol and a non-steroidal anti-inflammatory (NSAID) can be considered. Sedative premedication should be avoided.

It is best practice to induce anesthesia in theatre and not in an anesthetic room, thereby minimizing movement of the unconscious obese patient. Patients should be positioned in a 20 to 30 degree head-up position at induction, with sufficient support behind the shoulders and head to optimize intubation conditions [8]. Positioning the arms out laterally on arm supports is often helpful, both in terms of optimizing anesthetic access and allowing the surgeon easier access to the abdomen. Attention to pressure points is essential in the morbidly obese. Placement of an inflatable hover mattress to facilitate post-operative transfer is very useful. If a steep head-up posture is required, a footplate should be positioned to prevent sliding, and the patient should be securely strapped to the operating table.

Thromboprophylaxis should be instigated using compression techniques to cover the peri-operative period. The standard is intermittent pneumatic calf compression. Pre-operative low molecular weight heparins may be indicated in high-risk patients, and is recommended postoperatively in all cases. The ideal time for initial dose administration is probably 4 hours post-surgery, but this may be delayed in patients with marked surgical bleeding [9]. There is increasing evidence that thromboprophylaxis with low molecular weight heparins should be routinely continued for around 10 days postoperatively in all patients undergoing gastric bypass or other major procedures. In selected cases where patients are at very high risk or are unable to tolerate heparins, there may be a role for temporary inferior vena cava filter placement [10].

### Induction agents, neuromuscular blocking agents, and ventilation

The choice of induction agent depends on familiarity. The agents and technique used should be amenable to a stable induction that minimizes risk of desaturation.

There has been considerable debate regarding the timing of administration of neuromuscular blocking agents in cases where facemask ventilation is initially awkward. Traditional teaching advocated that when difficult intubation or ventilation is anticipated, administration of such neuromuscular blocking agents should be delayed until after adequate facemask ventilation has been achieved [11]. However, there is increasing consensus that facemask ventilation becomes easier once neuromuscular blocking agents have been given [12]. Difficult facemask ventilation is more common and much more serious in overweight and obese patients. Therefore, for patients in whom awake intubation is not felt to be indicated, neuromuscular blocking agents should be given early in the induction sequence.

The time between administration of the neuromuscular blocking agent and initiation of ventilation should be minimized in order to maintain adequate control. Facemask ventilation should be performed carefully to avoid distension of the stomach and bowel, and there should be a low threshold for the use of airway adjuncts, particularly a Guedel airway, to aid ventilation. A 20 to 30 degree head-up posture during induction facilitates ventilation in all patients, as well as assisting in intubation. When difficulties in facemask ventilation are anticipated, there may be an advantage in the use of higher-dose rocuronium or even suxamethonium to enable very early intubation.

Monitoring of neuromuscular blockade during surgery is advised. It is important to use a neuromuscular blocking agent with a predictable duration of action in order to avoid residual neuromuscular blockade during the recovery period. Common approaches include the use of agents such as atracurium, or an aminosteroid such as vecuronium or rocuronium. The longer duration of action and delay in offset of the aminosteroids is now counterbalanced by the availability of the γ-cyclodextrin reversal agent, sugammadex. Although currently not routine, the use of cyclodextrins to reverse neuromuscular blockade may have benefits and, in the future, may become more prevalent.

Maintenance of adequate relaxation for surgery is enhanced with remifentanil, and the use of this agent has the additional benefit of reducing volatile or propofol requirements.

### Rapid sequence induction

Rapid sequence induction (RSI) has historically been a common procedure in obese patients, and remains the technique of choice in patients with a history suggestive of active reflux. However, in patients without such a history, RSI is not required. A comprehensive review by Freid [13] concluded that RSI offered no benefit in fasted elective surgery patients who have no risk factors for reflux other than obesity or sleep apnea syndrome.

### Airway control

Patients who are morbidly obese have a reduced functional residual capacity and an increased basal oxygen utilization [14,15]. Therefore, once they become apneic, they experience rapid and early desaturation. Pre-oxygenation is mandatory as a standard of care, including in the head-up or ramped position, and is ideally performed using a tight-fitting facemask. Where this is not tolerated by the patient, an alternative technique should be used to achieve the best pre-oxygenation possible.

The most important consideration during induction of anesthesia and intubation is to maintain control of the airway. A technique should be used that minimizes the transition time from the airway being maintained by an awake patient to the airway being secured by the anesthetist. We advocate early use of a neuromuscular blocking agent, although in selected individuals there may be a role for awake fibre-optic intubation (FOI). Loss of airway control is most likely to occur in patients who are inadequately anesthetized and inadequately paralyzed.

There is a misconception that intubation in morbidly obese patients is much more difficult than intubation in non-obese patients. There is little evidence for this. Often, facemask ventilation and maintenance of adequate oxygenation is far more likely to be an issue. Several studies have shown that BMI alone is not a risk factor for difficult tracheal intubation, that is, the most severely overweight were not more difficult to intubate than other patients [8,16,17]. The strongest predictors of difficult intubation are: 1) male gender; 2) large neck circumference [8,18]; 3) limited neck mobility; 4) crowded mouth, as indicated by a high Mallampati score [8,18]; and 5) obstructive sleep apnea.

Reliable airway control is best achieved by: 1) Use of an appropriate relaxant and dose; 2) good positioning, including head elevation. ramping, and head-up tilt; and 3) help of a skilled assistant familiar with bariatric and airway issues.

FOI is seldom indicated, except for expected difficult intubation (similar considerations as for non-obese patients) or expected significant difficulty with facemask ventilation.

The role of airway adjuncts, such as the video laryngoscope, is under evaluation. Such airway adjuncts may prove extremely valuable, but at present, their use depends on the skills of the individual clinician. Principles of management of the ‘can’t intubate, can’t ventilate’ (CICV) scenario have been outlined by the Difficult Airway Society. These should be followed when a patient under general anesthesia with muscle relaxation cannot be intubated by direct laryngoscopy and mask ventilation is difficult/impossible [19].

### Maintenance

Options for maintenance techniques include either short-acting volatiles or total intravenous anesthesia (TIVA). The choice of technique is underpinned by a number of key principles.

#### Controllability

Because modern volatile agents have low blood-gas and oil-gas solubilities, their pharmacokinetics are only moderately influenced by obesity. However, the rapidity of onset and offset of anesthesia conferred by very low blood-gas solubility (for example, with sevoflurane or desflurane) is more marked in obese patients. Consequently, depth of anesthesia can be more rapidly titrated using these agents. In addition, volatile agents are titrated against an individual’s measured end-tidal concentration, whereas TIVA is titrated against an effect-site concentration derived from a population-based kinetic model. There is no widespread agreement on the kinetic model beyond 120 kg, and thus requires the anesthetist to use a degree of estimation when utilising propofol infusion techniques. Whenever TIVA is used, depth of anesthesia monitoring is recommended to facilitate titration of anesthesia.

#### Rapid wake-up: patient regains control of airway quickly

Recovery from anesthesia is often prolonged in obese patients [20]. These patients are also at increased risk of both aspiration and acute upper-airway obstruction after tracheal extubation [21]. These are rarely occurring complication, but are potentially catastrophic. Therefore, very rapid post-operative recovery is desirable in these patients to ensure early efficient coughing and to decrease the risk of post-operative respiratory complications.

A recent meta-analysis comparing short-acting volatile agents showed that desflurane reduced mean time to extubation by approximately 25% relative to sevoflurane. There was a further reduction in the variability of extubation time, with the group receiving desflurane having significantly fewer patients with a prolonged (15 minutes or longer) interval between the end of surgery and extubation [22].

Safe return of airway and swallowing reflexes has also been shown to occur more rapidly with short-acting volatiles. The time from anesthetic discontinuation to recovery of the ability to swallow was significantly shorter after desflurane anesthesia, and this effect was most marked in those patients with greater BMI and prolonged procedures [23].

#### Minimal respiratory depression

Anesthetics that allow for rapid emergence from anesthesia and rapid recovery of airway control tend to produce the least post-operative respiratory depression, therefore aiding carbon dioxide (CO_2_) clearance, restoration of deep breathing, and effective cough.

Inhalation anesthetics have a dose-dependent effect on respiratory drive and reflexes. In particular, they accentuate the depressant effects of opioids on the responses to hypoxia and hypercapnia. Previous work has shown that residual volatiles at the 0.1 minimum alveolar concentration may significantly depress the hypoxic drive in normal subjects, and these effects are likely to be exaggerated in patient who are morbidly obese. The ultra-short-acting volatiles that are the least metabolized have the smallest effect in depressing this hypoxic response [24]. Similarly, minimally metabolized agents do not share the issues of accumulation of volatile metabolites (such as compound A), and thus can be used at very low flow rates to maximize efficiency.

#### Minimal compromise of hemodynamic stability

All maintenance agents, both volatile and intravenous, cause vasodilatation and a reduction in blood pressure. With some maintenance regimens, this can be marked. In patients with reduced cardiac reserve, the use of agents with a degree of sympathetic activity may give a more stable peri-operative course, and could reduce the excessive use of fluids and vasopressor agents [25].

#### Early mobilization of patients after the procedure

The very rapid offset time and rapid awakening after maintenance with the ultra-short-acting volatile agents translates into earlier mobilization after surgery. Wherever possible, morbidly obese patients should be sat out in a chair by their bed later in the day of surgery. This is important for reducing post-operative complications, especially deep-vein thrombosis, but erect posture also appears to speed up the recovery of gut function and certainly has beneficial effects on post-operative respiratory mechanics. A key assumption of the Enhanced Recovery Programme, and widely accepted, is that early ambulation translates into earlier discharge [26].

### Extubation

The extubation technique used should aim to ensure that the transition from a controlled, intubated airway to a patient-protected airway and spontaneous ventilation is as quick, safe, and smooth as possible. Airway control around the time of extubation poses greater risks to overweight and obese patients than to lean patients.

The timing of extubation in these patients should be based on the balance of risk of loss of the airway versus patient comfort. Patients with significant respiratory co-morbidity will have to be almost fully awake and coughing on the tube before extubation can be allowed; however, this induce marked hypertension and cardiovascular stress. In order to allow slightly earlier extubation while minimizing the risks of failure, the following points should be observed.

•Use as little opioid as possible, or use short-acting opioids that clear rapidly, such as remifentanil.

•Minimize residual anesthetic at extubation.

•Ensure adequate reversal of neuromuscular blocking agents with the use of appropriate reversal agents (and confirm with a nerve stimulator if in doubt).

•Position the patient in an almost upright position to maximize respiratory mechanics; this may require lateral support with pillows to ensure the patient does not fall to one side.

•Consider using respiratory stimulants immediately after extubation to prevent hypoventilation and CO_2_ retention in patients likely to have an obtunded respiratory drive.

### Analgesia

One of the central tenets of the Enhanced Recovery Programme is that there should be a multimodal approach to immediate post-operative and peri-operative pain relief in order to reduce side effects of individual agents. Central to analgesia management of overweight and obese patients are the following points:

•Minimize the use of long-acting opioids.

•Use minimally invasive surgery with local anesthetic infiltration.

•Use appropriate dosing of non-opioid analgesics.

•The paracetamol dose given to overweight and obese patients should be similar to that of non-obese patients. However, clearance of paracetamol in overweight and obese patients is increased [27], therefore dosing may need to be more frequent.

•Use co-analgesic agents.

The post-operative pain reported by obese patients, and thus their analgesic requirement, is often less than expected [28]. This has been attributed to high motivation. Epidural analgesia is technically difficult, and may delay mobilization. This carries disadvantages for overweight and obese patients undergoing bariatric surgery, unless a large open wound is anticipated. Patient-controlled analgesia and epidural analgesia are rarely necessary, in part owing to increasing use of minimally invasive surgery and the availability of other analgesia options.

Paracetamol is a frequently used adjunct and has been shown to be markedly morphine-sparing in bariatric surgery. The risk of toxic metabolites from high-dose paracetamol is low, but in patients with known liver disease or who are in a malnourished state (rare in the morbidly obese population), the dose should adhere to the British National Formulary maximum of 4 g/day. Many centers use larger doses in very large patients, and doses of 6 g on the day of surgery are routine in some centers.

NSAIDs are effective as part of a multimodal regimen, but they should be used with caution in patients at high risk of post-operative renal dysfunction. If used judiciously, the potential bleeding risk of NSAIDs should be minimal. Traditionally, many clinicians have avoided NSAIDs in patients with a history of asthma. Aside from cases of known sensitivity, there is very little evidence to support this practice [29,30]. The use of diclofenac or ketorolac seems appropriate.

Gabapentin and pregabalin are frequently used as co-analgesics, but appear to be markedly sedating and, in the obese patient who needs to awaken rapidly and protect their airway, this side effect may be unacceptable [31].

Dexmedetomidine has just become available in the UK, and it appears to be a useful co-analgesic agent, especially in obtunding sympathetic responses. The roles of ketamine and lidocaine infusions are under investigation. These might prove useful in the future.

### Anti-emetics

Post-operative nausea and vomiting (PONV) is a common complication of general anesthesia, which is extremely unpleasant for the patient and can lead to increased recovery-room time and extended hospital stay. Manipulation of the upper stomach, such as occurs in bariatric surgery, is associated with a significant incidence of nausea, thus good anti-emetic therapy is highly desirable [32].

Guidelines on PONV published in 2003 recommended 5-hydroxytryptamine 3 (5-HT3) receptor antagonists as first-line preventive agents in patients categorized as high risk [33]. Ondansetron is the most frequently used, but its relatively short duration of action means that this drug should be given towards the end of the procedure. In procedures and patients with a high risk of PONV, use of multiple anti-emetics is recommended, and the combination of a 5-HT3 antagonist (for example, ondansetron) and dexamethasone is a frequently chosen pairing [34]. There is further benefit to be gained with the use of triple anti-emetic therapy; additional agents commonly added include cyclizine and metoclopramide.

Nitrous oxide (although rarely used in UK bariatric surgery) has been implicated as a risk factor for PONV, and should probably be avoided. Likewise, opioid use should be kept to a minimum. In patients with a strong history of PONV, use of a continuous infusion of propofol may be indicated.

The contribution of nausea to the delay in patient mobilization and patient discomfort is very important, and the best anti-emetic regimen is still to be determined. The role of other anti-emetic therapies, including hyoscine patches or pressure-point techniques, may be considered. It is important to remember that nausea may be a symptom of hypotension and of hypoglycemia in patients with diabetes.

### Priorities for further clinical research

There is a pressing need for further clinical research in the management of this challenging patient group. Although much has been published on the underlying molecular mechanisms of obesity, the way in which these processes relate to pathophysiology of the disease and, in particular, how they affect anesthesia and the peri-operative course is poorly understood. We recommend a program of structured research covering: 1) mechanistic investigation: molecular and pathophysiologic mechanisms to affect peri-operative medicine; 2) potential disease response modifiers; and 3) a program of translational research to bring the bench to the bedside.

We suggest the following areas as priorities for clinical research.

•Identification of how co-morbidities significantly affect the peri-operative course, for example, heart failure and ischemic heart disease; and particularly sleep apnoea / anaesthetic agent interactions and the increased risk of post-operative respiratory depression.

•Design of anesthetic strategies to minimize risk of peri-operative morbidity, particularly nausea.

•Improvement of tests that predict difficult intubation.

•Definition of the role of co-analgesic agents in reducing opioid requirements and discomfort.

## Conclusions

In this consensus statement, we have addressed the clinical issues that anesthetists are increasingly likely to encounter, given the rising prevalence of obesity in the UK. We have emphasized the following key principles for best practice.

•Better outcomes may be achieved by awareness of the key issues that may arise in this patient population.

•The anesthetist should maintain control of the patient and minimize periods of potential risk or instability.

•Techniques that are familiar, reproducible, and safe should be used in preference to unfamiliar techniques that may enhance the risk to the patient.

•Anesthetic techniques should be used that encourage better outcomes, early mobilization, and shortened length of hospital stay.

These guiding principles align with enhanced surgical recovery.

Given the correct care, anesthesia of overweight and obese patients is straightforward. However, problems have greater potential to escalate with this patient population than with other patient populations. With an understanding of the issues addressed in this consensus statement, and with appropriate use of techniques and agents, these patients can be managed as safely as patients of normal weight.

## Abbreviations

5-HT3: 5-hydroxytryptamine 3; AAGBI: Association of Anaesthetists of Great Britain and Ireland; CICV: Can’t intubate can’t ventilate; FOI: Fibreoptic intubation; NHS: National Health Service; NSAID: Non-steroidal anti-inflammatory drug; PONV: Post-operative nausea and vomiting; RSI: Rapid sequence induction; SOBA: Society for Obesity and Bariatric Anaesthesia; TIVA: Total intravenous anesthesia; WHO: World Health Organization.

## Competing interests

In May 2010, Baxter convened a panel of experts to discuss the clinical applications of Suprane. This consensus document is the result of that meeting and consultation with the Society for Obesity and Bariatric Anaesthesia (SOBA). KA, OB, MB, and AP have received payments from Baxter for consultancy work.

## Authors’ contributions

All authors read and approved the final manuscript.

## Expert panel

The members of the expert panel are: Euan Shearer, Consultant Anesthetist, University Hospital Aintree, Liverpool, UK; Andrew Paix, Consultant Anesthetist, Princess Royal University Hospital, Orpington, UK; Claire Nightingale Consultant Anesthetist, Wycombe Hospital, High Wycombe, UK; Nick Kennedy, Consultant Anesthetist and Intensivist, Musgrove Park Hospital, Taunton, UK; Andy Kendall, Consultant Anesthetist, St Richard’s Hospital, Chichester, UK; William Fox, Consultant Anesthetist Royal Cornwall Hospital, Truro, UK; Jonathan Cousins, Consultant Anesthetist, Charing Cross and The Hammersmith Hospitals, London, UK; Olumuyiwa Bamgbade, Consultant Anesthetist, Manchester Royal Infirmary, Manchester, UK; and Kamran Abbas, Consultant Anesthetist, Manchester Royal Infirmary, Manchester, UK.
